# Primary Site of Coxsackievirus B Replication in the Small Intestines: No Proof of Peyer’s Patches Involvement

**DOI:** 10.3390/microorganisms9122600

**Published:** 2021-12-16

**Authors:** Shubhada Bopegamage, Katarina Berakova, Pavol Gomocak, Renata Baksova, Jochem Galama, Heikki Hyoty, Sisko Tauriainen

**Affiliations:** 1 Enterovirus Laboratory, Institute of Microbiology, Faculty of Medicine, Slovak Medical University, Limbova 12, 833 03 Bratislava, Slovakia; shubhada.bopegamage@szu.sk or; 2 Martinske Biopticke Centrum s.r.o., V. Spanyola 47A Street, 010 01 Zilina, Slovakia; katarinaberakova@gmail.com; 3 Immunohistochemical Laboratory, Medical Laboratory Pathology and Cytology, Cytopathos, s.r.o., Kutuzovova 23, 831 03 Bratislava, Slovakia; gomolcak@cytopathos.sk; 4 Laboratory of Human Pathogens, Institute of Microbiology, Faculty of Medicine, Slovak Medical University, Limbova 12, 833 03 Bratislava, Slovakia; renata.baksova@szu.sk; 5 Radboud University Medical Centre, Department of Medical Microbiology, 6525 GA Nijmegen, The Netherlands; jmdgalama@gmail.com or; 6 Faculty of Medicine and Health Technology, Department of Virology, Tampere University, 33100 Tampere, Finland; heikki.hyoty@tuni.fi; 7 Fimlab Laboratories, Pirkanmaa Hospital District, 33521 Tampere, Finland; 8 Department of Biomedicine, University of Turku, 20520 Turku, Finland

**Keywords:** coxsackieviruses, small intestines, Peyer’s patches, mice

## Abstract

Background: Enterovirus (EV) infections are associated with a broad range of diseases. Since the first experimental infection of primates with poliovirus (PV), tonsils and the Peyer’s patches (PPs) have been believed to be the primary replication sites of EVs. Our aim was to localize different viral markers in the small intestines (SI) of coxsackievirus B (CVB) orally and intraperitoneally (i.p.) infected mice. Methods: Transverse sections of SIs of both infected and control male outbred mice were collected at different intervals post-infection (p.i) and analyzed for presence of interferon-alpha (IFN-α) and viral protein VP1 by immunohistochemistry and in situ hybridization (ISH). Fluorescent marker, eGFP, was identified in cryosections of mice infected with eGFP-CVB3. Results: In the infected SIs, we observed enlarged germinating centers (GCs) in the PPs; IFN-α was detected in the PPs and mucosal layer of the SIs. However, VP1, viral RNA and the eGFP were absent in the GCs of PPs at all stages of infection irrespective of the virus strains used. Conclusions: Virus was present in the epithelial cells but not in GCs of the PPs of the murine SIs. Our results do not support the hypothesis of EV replication in the PP especially in the GCs.

## 1. Introduction

Genus *Enterovirus* belongs to the family *Picornaviridae*, it is classified into fifteen species of which seven (*Enterovirus-A*, *-B*, *-C* and *-D*, and *Rhinovirus-A*, *-B* and *-C*) are human pathogens. These viruses are transmitted by the fecal-oral and respiratory routes. Enteroviruses (EVs) are common around the world and cause a variety of diseases in humans, ranging from mild and asymptomatic to severe life threatening central nervous system (CNS) and systemic infections. Young children are the most affected. After the introduction of poliovirus (PV) vaccines, enterovirus-71 (EV-71) and enterovirus-D68 (EV-D68) have caused the most concern. EV-71 has caused epidemics in children in the Asia–Pacific region, with severe CNS infections, paralysis and fatalities. EV-D68 caused outbreaks with severe lower respiratory infections. Coxsackieviruses (CVs) can cause severe diseases, such as aseptic meningitis, encephalitis, myocarditis, and pancreatitis. Especially the serotype CVB4 has been suggested to have a role in the development of type 1 diabetes.

Peyer’s Patches (PPs) are a group of lymphoid follicles of the lymphatic system situated in the mucoidal membrane lining of the small intestines (SIs), often located in the ileum. The PPs are a part of the immune control system for recognition and destruction of the pathogens. The EVs spread by the fecal-oral route, they overcome the acidic environment of the stomach and bile and then reach the SIs. They replicate in the SIs for 2–8 weeks [1] during which fecal shedding of the virus has been reported. It is still not clear where exactly the viruses replicate in the intestine.

Chimpanzees were used to study poliovirus infections in the early studies. Since the observation by Bodian et al. [2], the primary replication of all EVs has been believed to be the PPs. These authors demonstrated high viral titers in the PPs of the SIs of chimpanzees, orally infected with PV. Additionally, they showed that virus titers were absent in the rest of the ilium. The PVs are the most studied EVs. Although the *EV* genus contains a diverse group of viruses which use different receptors for entry, most results of the PV studies are applied for other EVs. Despite the recent improvement and technical modernization during last few decades, the involvement of PPs in the infection cycle of EVs has not been extensively studied or questioned after the initial studies from the late 1950s.

Genetically engineered mice models expressing the human receptor for PVs have been used to study the viral pathogenesis since the time they were created in the beginning of 1990s [3,4]. Engineering the expression of the human receptor to poliovirus (hPVR) made these mice susceptible to PVs injected via different routes (intravenous, intramuscular, intrathecal and direct injection into the spinal cord) [3,5,6]. This model has since replaced the primate model.

The mouse coxsackie-adenovirus receptor (mCAR) is similar to the human coxsackie-adenovirus receptors (hCAR) [7] making mice susceptible to CV infections. Mice have been used as experimental models for studying the CV pathogenesis. The CVs belong to the genus *EV* as do the PVs and their pattern of spread in body of a primate is believed to be similar to that of PVs.

Our aim, therefore, was to detect presence of the viral markers by different methods in the SIs of CVB infected mice after oral and intraperitoneal (i.p.) infection with a focus on the PPs. We present data from three earlier experimental study series where we observed the absence of viral markers in the PPs.

## 2. Materials and Methods

Mice: CD1 and Swiss albino male mice (outbred) were obtained from Faculty of Medicine Masaryk University, Brno, Czech Republic. The mice were maintained in the specific pathogen free (SPF) Laboratory Animal Facility of the Slovak Medical University in Bratislava and had free access to food and water, with controlled room temperature (22 ± 0.5 °C), automatic light, reverse 12/12 h light/dark cycle and humidity (55% ± 5%). Mice were caged 2 or 3 per cage with sterile bedding, water and commercial food pellets (Top dovo, Trnava, Slovak Republic).

Viruses: CVB3-Nancy, laboratory strain [8]; CVB4-JVB, laboratory strain (both from from Institute of Hygiene and Epidemiology, (known as USOL), Prague, Czech Republic) [9], and a recombinant eGFP-CVB3 virus expressing an enhanced green fluorescence protein (eGFP) which acts as a marker protein a kind gift from Prof. Andreas Henke, Jena, Germany with the permission of R. Feuer and L. Whitton [10].

### 2.1. Mice Infections and Collection of Organs

Swiss albino mice (Velaz s.r.o., Prague, Czech Republic) and CD1 mice (Envigo Innovative ++++, Udine, Italy), 3–4 weeks of age, were infected by oral or (i.p.) routes, as described by us previously [8]. The experimental mouse study and protocols were reviewed and approved by the Institutional Animal Ethics Committee of the Slovak Medical University followed by State Veterinary and Food Control Authority of the Slovak Republic.

Organs: SIs were collected at different times (described below in each set of experiments). Pieces of the small intestines 4 cm in length, (about 4 cm from the appendix) were cut and fixed in 4% formaldehyde. The SIs were selected from three sets of experiments, as shown by the color markings in Supplementary Table S1. The control groups were age and sex matched, and received 0.5 mL of PBS as mock infection. These mock infected controls groups were included in all the three experiments parallel I, IIa and IIb, III. 3 mice were sacrificed for each time point parallel to the infected mice. Organ samples used in this study are presented in Supplementary Table S1.

Set (I): Swiss albino mice were infected via oral and i.p. routes with CVB3-Nancy using four different doses: 5 × 10^3^, 5 × 10^5^, 5 × 10^7^ and 5 × 10^9^ TCID_50_. The mice were sacrificed at days 3, 7, 10, 14, 21, 28, 35, 48, 56, 63, 98 and 147 post infection (p.i.), details described in our earlier work [8]. Three mice were sacrificed per time interval as stated above.

Set (IIa): Swiss albino and Set IIb CD1 mice were infected via oral and i.p. routes with CVB4-JVB, with a dose of 5 × 10^7^ TCID_50_. Small intestines were collected from mice sacrificed at different hours p.i.: 2, 4, 6, 8 and 24 h. Three mice sacrificed per time interval. Pieces of the SIs were snap frozen in tubes for PCR or fixed in formaldehyde and embedded in paraffin. Small intestine tissue sections were stained with haematoxylin eosin and VP1 staining.

Set (III): CD1 mice were infected with eGFP-CVB3 at a dose of 5 × 10^7^ orally and i.p. The SI s were collected from mice sacrificed at different hours p.i.: 2, 4, 6, 8 and 12 h, 24 h. Three mice sacrificed per time interval. Pieces of the small intestines were fixed in formaldehyde and embedded in paraffin or straightened out and snap frozen in cryotubes and stored frozen at −80 °C.

### 2.2. Immunohistochemical Analysis

Immunohistochemical (IHC) analysis was used to detect the EV capsid protein, VP1, and interferon-alpha (IFN-α) in small intestine samples. VP1 detection was done by two methods in two labs, both using the Monoclonal Mouse Anti Enterovirus antibody against the VP1 protein (Clone 5-D8/1, Dako). The primary and secondary antibodies were carefully titrated using infected control tissues. Initially all sections of the SIs of the mock infected control and infected mice were analyzed. Samples and methods used for detection are presented in Supplementary Table S1.

VP1 Method 1 [8,9]: This method was used as the primary method for detection in SI tissues of all the mice from all the three sets (groups I, II and III with respective controls). The steps for processing the SI tissues i.e., antigen retrieval, (0.1 M sodium citrate buffer, microwave at 750 W, 2 times for 5 min), quenching, (0.3% hydrogen peroxide for 15 min. at room temperature), and background blocking (protein Serum Free Block [Dako Cytomation] for 20 min) were followed by exposure of the processed tissue section to the primary antibody. Dilutions of the antibodies were chosen after standardization using different dilutions and varying incubation times of the primary antibody Monoclonal Mouse Anti-Enterovirus antibody anti-VP1 (5-D8/1 Dako, Næstved, Denmark) for 60 min. The chosen 1:250 dilution was mixed with Biotinylation Reagent to bind secondary antibody and incubated for 15 min (reagents from DAKO ARK Kit Animal Research Kit). The Blocking Reagent was then added to the mix and incubated for 5 min to bind residual Biotinylation Reagent. This step minimized the potential interaction with endogenous immunoglobulin in the specimen. The biotin labeled primary antibody was applied to the specimens and incubated for 15 min. After washing 3 times with phosphate buffered saline (PBS), the specimens were incubated with streptavidin-peroxidase and again washed in PBS. A freshly prepared chromogen/substrate reagent solution (diaminobenzidine/hydrogen peroxidase or aminoethycarbazol/hydrogen peroxidase) was applied. The chromogen substrate was incubated for 3 min to appropriate intensity. Slides were rinsed 3 times to stop the staining.

The immunostained tissue sections of small intestine were counterstained with haematoxylin for 10 s. The reaction was stopped by placing the slides under direct tap water.

VP1 Method 2: Selected tissues as seen in Supplementary Table S1 were analyzed by method 2. This method was used for confirming the results of method 1. The anti-VP1 (5-D8/1 Dako, Næstved, Denmark) was used at a dilution of 1:1000 using the EnVision+ polymer technique and a TechMate™ 500 Immunostainer (Dako Cytomation Næstved, Denmark A/S). Antigen retrieval was performed on rehydrated sections in a microwave oven at 850 W for two 7-min cycles using Tris- EDTA buffer (pH 9.0) as the retrieval solution. Diaminobenzidine (DAB) was used as a chromogen and hematoxylin as a nuclear stain. The specificity of immunohistochemistry was controlled by omitting the primary antibodies or replacing them with irrelevant anti-sera. Known positive tissue samples from enterovirus-infected mice were used to confirm the staining reliability of all separate staining batches [11].

IFN-α immunohistochemical analysis: We used the polyclonal rabbit antibody against mouse IFN-α (Anti- Mouse Interferon Alpha, Rabbit Serum, Piscataway, NJ, 08854, USA, PBL). We followed the method described by us previously [8].

Only SIs of infected and control mice of Set (I) experiment were tested for presence of IFN-α.

### 2.3. In Situ Hybridization

Selected tissues as seen in Supplementary Table S1 were analyzed by this method. Immunohistochemical analysis of VP1 protein localization was confirmed by in situ hybridization detecting viral RNA using an EV specific detection probe designed against the conserved 5′noncoding region detecting all enteroviruses (sequence GAA ACA CGG ACA CCC AAA GTA GTC GGT TCC GCT GCR GAG TTR CCC RTT ACG ACA). The probe was 3′ end-labeled with digoxigenin (DIG) using the DIG oligonucleotide tailing kit (Roche Diagnostics Ltd., Welwyn Garden City, UK). Ten pmol of the probe was used for one labeling reaction. Amount of probe in the hybridization cocktail was 250 ng, and the used hybridization time was 3 h. Binding of the probes was documented by anti-DIG antibody, which was conjugated with alkaline phosphatase. This enzyme, together with its substrate nitroblue tetrazolium/bromo-chloro-3-indolyl-phosphate (NBT/BCIP), yields an insoluble purple precipitate, which can be visualized using a light microscope. Known positive and negative tissue samples from enterovirus-infected and non-infected mice were used as controls in all separate in situ hybridization experiments. The hybridization was performed as published earlier [11,12].

### 2.4. Detection of eGFP-CVB3 in Tissues

Cryosections (5 µm) of the frozen organs of the eGFP-CVB3 infected mice (set III) were placed on Superfrost plus slides. The tissues of SIs were fixed on the slide by immersing the slides in precooled acetone (−20 °C), slides were dried and examined directly under fluorescent microscope Olympus BX60, Santa Barbara, CA, USA.

## 3. Results

Outbred male mice were infected via oral and i.p. route with CVB4-JVB, CVB3-Nancy and eGFP-CVB3. Mice were sacrificed at different times post infection. Transverse sections of the small intestines were selected for analysis. We analyzed the presence of viral markers (VP1 protein or RNA) and IFN-α by different methods.

### 3.1. Activation of PP’s Germinating Centers

**Figure 1:**
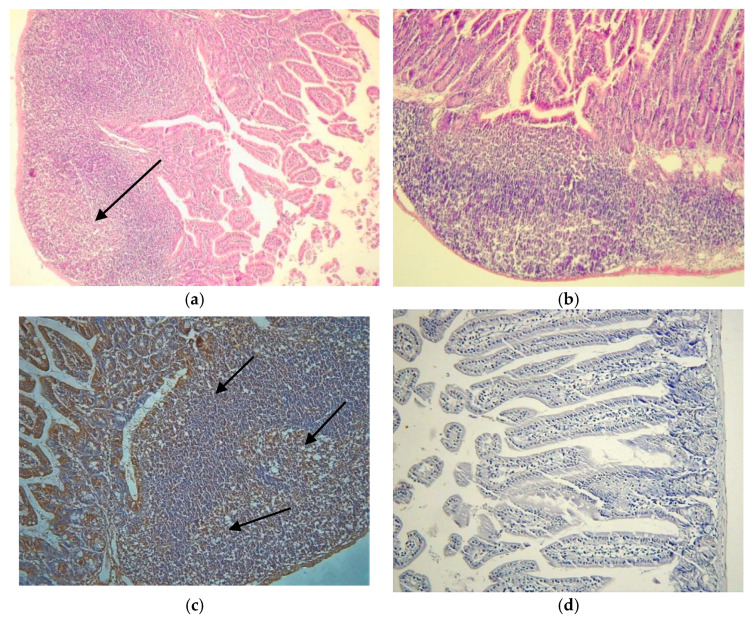
Peyer’s patches (PP). (**a**) Hematoxylin eosin staining of a typical PPs with enlarged germinating centers at day 7 post infection (p.i.) of orally infected mouse with CVB3-Nancy, Set 1 (20×); (**b**) normal PP of the mock infected control mouse at day 7 p.i. of an orally infected mouse, Set 1 (20×); (**c**) Localization of IFN-α in the PP, the brown staining, by immunohistochemical analysis (40×), Set (I) at day 3 p.i. (40×); (**d**) Small intestine sample of mock infected control mouse from Set I at day 3 p.i. (20×).

The PP’s showed activated germinating centers (GCs) after CVB infections (Figure 1a), the number of activated or enlarged PPs were increased after oral infection as compared to the control (Figure 1b).

IFN-α production was induced in the PPs after infection as shown by immunohistochemical analysis. IFN-α was localized in the GCs of the PP and in the sub-epithelial dome (SED) area (Figure 1c). The control is shown in Figure 1d.

### 3.2. Localization of Viral Markers

Localization of virus was determined by two different immunohistochemical methods, both using the same primary antibody (monoclonal anti-EV VP1, Clone 5D8/1, Dako, in combination with the ARK in method 1 and using the EnVision+ polymer technique and a TechMate™ 500 Immunostainer in method 2) and by in situ hybridization. Results of the VP1 staining of all sections of the SIs of infected mice by method 1 and 2 are presented in Supplementary Table S1. Selected SI samples were stained by IHC method 2 for VP1 and by in situ hybridization to confirm the staining results of method 1.

**Figure 2:**
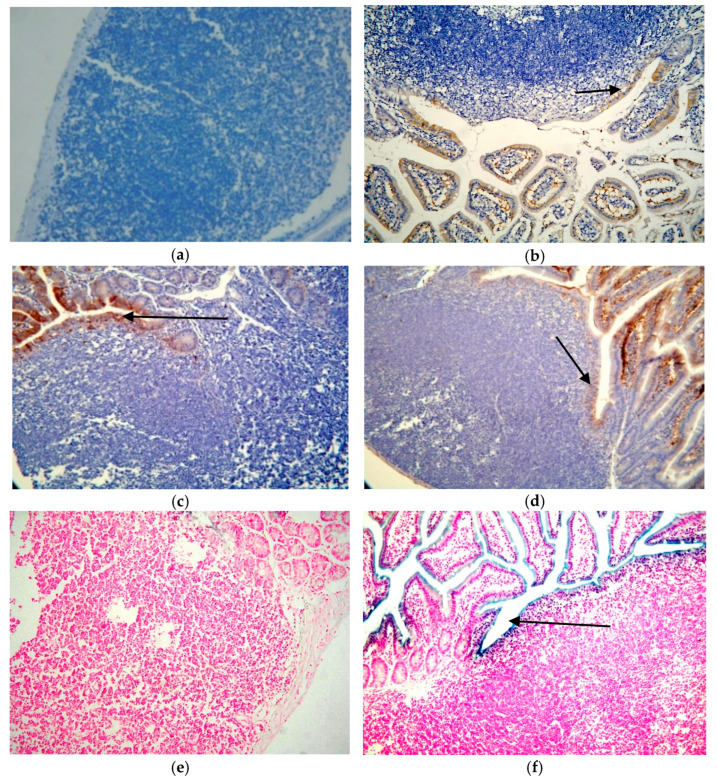
Localization of viral markers in mouse SI tissue sections after CVB infection by immunohistochemical analysis and in situ hybridization. (**a**) VP1 staining of the SI by method 1 of mock infected control (PBS) (40×); (**b**) SI of orally infected mouse (CVB4-JVB) of Set IIa mice, 4 h p.i. by method 1 (40×); (**c**) tissue section of SI of Set I (CVB3) infected mice stained by using method 2, SI of orally infected day 7 p.i. Arrows show the positivity in the FAE region (40×); (**d**) SI of i.p. infected mouse, day 7 p.i. (40×) (**e**) in situ hybridization of the SI tissue of the control (PBS) (40×), day 3 p.i. after oral infection; (**f**) orally (CVB3 infection) infected mouse of Set I mice 3 days p.i. (60×).

Figure 2a–f, show the results of the analysis of the tested viral markers by methods 1 and 2 and of the ISH. The VP1 protein were absent in the PPs in infected mice by both routes and by methods 1 and 2 used and EV RNA by ISH. The VP1 protein was localized in the follicle-associated epithelium (FAE) the lumen villi and in a few cells of the SED area already at 4h p.i. CVB4-JVB oral infection (Figure 2b and Supplementary Table S1). Viral RNA was localized in the villi and mainly in the FAE area similar to the VP1 staining (Figure 2f). PPs were clearly free of the viral markers at all time points, despite the infection route with both detection methods.

The VP1 positivity continued until day 21 p.i. in the intestines of mice infected with CVB4-JVB and those with CVB3-Nancy. The smooth muscle cells showed positivity at day 63 p.i. and day 98 p.i. confirming our previous results [8,9], Supplementary Table S1.

### 3.3. Detection of eGFP-CVB3 in Tissues

We followed the eGFP marker by fluorescent microscopy in orally and i.p. eGFP-CVB3 infected mice. The presence of eGFP was observed as bright fluorescing regions or spots.

**Figure 3:**
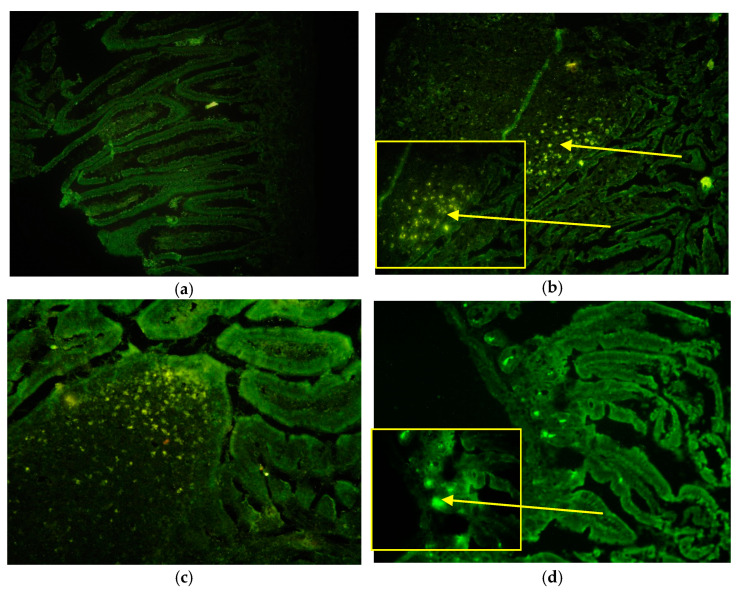
Localization of eGFP-CVB3 in the small intestines of infected mice. The fluorescent signal of enhanced green fluorescent protein (eGFP) can be seen as bright yellowish green fluorescent. The bright yellow arrows show the positive signals of the eGFP positive infected areas. (**a**) absence of fluorescence at 4h in the mock infected control mice, 20×; (**b**) At 6 h p.i. fluorescence can be seen in the sub-epithelial dome area of the PP and the FAE area after oral infection 20× with zoomed in 40×; (**c**) At 12 h p.i. fluorescence can be seen in the dome area of the PP and the FAE area after i.p. infection 20×; (**d**) at 8 h p.i. eGFP can be seen in the base of the villi or crypts of Lieberkuhn 20× and zoomed in at 40×. The yellow arrows show positive signals.

Figure 3a shows control SI. In the infected mice fluorescence was seen in the tip of the PPs in the SED and FAE regions and in the lumen in the oral route of infection (Figure 3b,c). The fluorescence appeared in the villi by 6 h in both the routes of infection (Supplementary Table S1). In both routes of infection, the virus appeared also in the crypts of Lieberkuhn at the base of the villi is just above the smooth muscle lining (Figure 3d). In contrast to the CVB3 and CVB4 virus infections, in these mice the fluorescence could be observed until 24 h p.i. Fluorescence signal was absent in mock infected mice SI samples.

## 4. Discussion

In our study, we have localized the viral markers in the SI and followed up the PPs. We noted the presence of IFN-α in the GCs after 3 days p.i. On the other hand, our results show clear absence of virus markers (viral RNA, VP1 and eGFP) in the GCs of the PPs of the mouse intestine after oral and i.p. infection. Considering the overall results, we have shown that the gross stroma of the PPs does not show presence of the viral markers even after 24 h post infection although IFN-α is induced confirming our findings [8]. The CVBs are known to induce IFN-α. The absence of virus in the GCs contrasts the findings of others who showed that, upon i.p. infection, coxsackievirus preferentially infects B lymphocytes within the gut-associated lymphoid tissue [13,14,15]. Activation of PPs GCs was only seen in the infected mice. Yet we should consider the limitation to the observation. Impulse of intake of food and the microbiome which consist of numerous antigens also lead to activation of the PPs.

The primary monoclonal VP1 antibody, Clone 5D8/1, used in the study has been shown to have cross reactivity and to react in some studies with non-infected tissues. We have eliminated the possibility by optimalization and standardization steps. We have therefore included the ISH as a confirmative method. The mock infected mice and negative control tissues did not show positivity by both IHC methods.

The virus was localized in the eGFP-CVB3 infected mice in the epithelial cells of the FAE which are localized in the dome of the PPs. The M cells are known to be specialized epithelial intestinal cells present in the FAE internalize particulate antigens and help in inducing the immune responses to pathogens [16]. The FAEs lie towards the lumen side of the SIs and contain blood vessels through which the virus spreads. Our results show that the viral markers were clearly present in the FAE region of the PPs. The viral markers were in the epithelial cells of the lumen in the first 24 h. The 3 days p.i. also showed positivity as observed by us previously [8]. The microfold (M) cells are located along the FAE. We have not used a specific staining for tagging the M cells. At this point we may only hypothesize that the virus may be present in the M cells. Therefore, we can only suggest that the CVBs replication may take place in the M cells.

After oral infection of the virus at 6h p.i. we also observed the presence of viral markers in the dome region below the epithelial lining the sub-epithelial dome (SED) and a few cells directly below the SED. This region is known to consist of the follicular area rich in 3 types of antigens presenting cells the memory B cells, macrophages (MØ), and dendritic cells (DC) [17,18]. Yet in contrast to these studies of Khan et al. [19] the CD155 which is the receptor in these transgenic mice showed moderate expression in the subepithelial dome, FAE and interfollicular cell regions of the PPs in these mice and absence of the receptor in the GCs but in the tunica muscularis.

Based on our results we suggest that the primary infection is not in the PPs. We also take the liberty to hypothesize that the primary spread is possibly via the M cells and epithelial cells of the lumen, which are closely in contact with the blood vessels, and not via the B lymphocytes. The virus then disseminates to secondary lymphoid organs via the blood to the spleen and other lymphoid organs but not in the first few hours. However, further studies are needed to confirm this hypothesis.

## Data Availability

The data presented in this study are available on request from the corresponding author.

## Supplementary Materials

The following are available online at https://www.mdpi.com/article/10.3390/microorganisms9122600/s1, Table S1: Number of small intestines showing positivity in the villi or smooth muscles in the infected mice by using different methods for localizing viral markers at different time points.
