# Supplementary material for: Primary Site of Coxsackievirus B Replication in the Small Intestines: No Proof of Peyer’s Patches Involvement
Source: Microorganisms. 2021 Dec 16;9(12):2600. doi: 10.3390/microorganisms9122600 (PMC8709031; doi:10.3390/microorganisms9122600)
Supplement: Supplementary file 1 [file microorganisms-09-02600-s001.zip › microorganisms-1185696-supplementary.pdf]

Supplementary Table S1: Number of small intestines showing positivity in the villi or smooth muscles in the infected mice by using different methods for localizing viral markers at different time points.

		Doses and routes of Infection							
Details of the infections	Days p.i.	5 x 10^3		5 x 10^5		5 x10^7		5 x 10^9	
		**ip**	**oral**	**ip**	**oral**	**ip**	**oral**	**ip**	**oral**
	**Set I**								
**Set I** Swiss albino male mice infected with CVB3-Nancy 5 x 10^3, 5 x 10^5, 5 x10^7 and 5 x 10^9 $TCID_{50}$.	3	3/3*	3/3	3/3	3/3	3/3	3/3	3/3	3/3
	7	3/3	3/3	3/3	3/3	3/3	3/3	3/3	3/3
	10	3/3	3/3	3/3	3/3	3/3	3/3	3/3	3/3
	14	1/3	3/3	3/3	3/3	3/3	3/3	3/3	3/3
	21	1/3	3/3	1/3	3/3	3/3	3/3	3/3	3/3
	28	0/3	3/3	0/3	3/3	1/3	3/3	1/3	3/3
	35	0/3	0/3	0 /3	1/3	0/3	1/3	0/3	2/3
	48	0/3	0/3	0/3	0/3	0/3	0/3	0/3	0/3
	56	0/3	0/3	0/3	0/3	0/3	1/3	0/3	1/3
	63	0/3	0/3	0/3	0/3	0/3	2/3	0/3	2/3
	98	0/3	0/3	0/3	0/3	0/3	0/3	0/3	1/3
	147	0/3	0/3	0/3	0/3	0/3	0/3	0/3	0/3
	**Set II a**								
**Set II a** Swiss albino male mice infected with CVB4-JVB, dose of 5 x10^7 $TCID_{50}$.	2h	ND°	ND	ND	ND	0/3	1/3	ND	ND
	4h	ND	ND	ND	ND	0/3	2/3	ND	ND
	6h	ND	ND	ND	ND	1/3	3/3	ND	ND
	8h	ND	ND	ND	ND	1/3	3/3	ND	ND
	24h	ND	ND	ND	ND	1/3	3/3	ND	ND
	**Set II b**								
**Set II b** CD1 ale mice infected with CVB4-JVB, dose of 5 x10^7 $TCID_{50}$.	2h	ND	ND	ND	ND	0/3	1/3	ND	ND
	4h	ND	ND	ND	ND	1/3	2/3	ND	ND
	6h	ND	ND	ND	ND	1/3	2/3	ND	ND
	8h	ND	ND	ND	ND	1/3	3/3	ND	ND
	24h	ND	ND	ND	ND	1/3	3/3	ND	ND
	**Set III**								
**Set III** CD1 male mice infected with eGFP-CVB3 at a dose of 5 x10^7 $TCID_{50}$.	2h	ND	ND	ND	ND	0/3	1/3	ND	ND
	4h	ND	ND	ND	ND	1/3	2/3	ND	ND
	6h	ND	ND	ND	ND	1/3	1/3	ND	ND
	8h	ND	ND	ND	ND	1/3	2/3	ND	ND
	24h	ND	ND	ND	ND	2/3	3/3	ND	ND

Mock infected control tissue results were not shown as they were negative. In all sets, 3 mice were used at all time points, as mock infected controls.
*= 3/3 shows 3 positive tissues, each from a separate mouse/total number of mice
° = ND Not Done indicates which virus dosed were not used for infections.

Selected for confirmation with VP1 staining by Methods 1 and 2 only.

Selected for confirmation with VP1 staining by Methods 1, 2, and *In situ* hybridization.

Done only by Method 1, RT-PCR or eGFP analysis.
